# Biomarker Guided Diagnosis of Septic Peritonitis in Dogs

**DOI:** 10.3389/fvets.2019.00208

**Published:** 2019-06-27

**Authors:** Pia Martiny, Robert Goggs

**Affiliations:** College of Veterinary Medicine, Cornell University, Ithaca, NY, United States

**Keywords:** sepsis, biomarker, effusion, gradient, lactate

## Abstract

Septic peritonitis (SP) is common in dogs and is associated with high mortality. Early recognition is essential to maximizing survival and may be aided by biomarker measurement. The present study aimed to evaluate the ability of biomarkers to discriminate septic peritonitis from non-septic ascites (NSA). Eighteen dogs with SP and 19 age-matched controls with NSA were enrolled. Contemporaneous blood and peritoneal effusion samples were obtained. Concentrations of cell-free DNA (cfDNA), cytokines, glucose, lactate, N-terminal pro-C-type natriuretic peptide (NT-proCNP), nucleosomes, and procalcitonin (PCT) were measured using commercial reagents and assays. Paired biomarker concentrations were compared with the Wilcoxon matched-pairs signed rank test, and biomarker concentrations between groups were compared with the Mann-Whitney *U*-test. *P*-values were adjusted for multiple comparisons using the Bonferroni correction. Receiver operating characteristic curves were generated to assess the ability of the above biomarkers to discriminate SP from NSA. Dogs with SP had significantly greater blood CCL2 concentrations than dogs with NSA (*P* = 0.032). Dogs with SP had significantly greater effusion CCL2, IL-6, IL-10, and lactate concentrations than dogs with NSA (*P* ≤ 0.0121). Blood-effusion concentration gradients of CCL2, glucose, IL-6, IL-10, and lactate were significantly different in dogs with SP compared to dogs with NSA (*P* ≤ 0.0165). Effusion lactate concentration had the highest AUROC value (0.866, 95% CI 0.751–0.980, *P* = 0.0001), although other biomarkers performed similarly. An effusion lactate concentration of 4.2 mmol/L was 72.2% (95% CI 46.5–90.3%) sensitive and 84.2% (95% CI 60.4–96.6%) specific for the diagnosis of SP.

## Introduction

Septic peritonitis (SP) is inflammation of the peritoneal cavity caused by the presence of viable bacteria, and is a surgical emergency (1). The diagnosis of SP can be made by cytologic identification of intracellular bacteria in peritoneal fluid, by positive bacterial culture of peritoneal fluid, and through gross identification of gastrointestinal leakage at the time of laparotomy (2–4). Early diagnosis is crucial to enable timely and definitive treatment. Case fatality rates of 36–67% (2001–2018) are reported in dogs with SP (2–6). Diagnosis can be challenging when bacterial numbers are low, such as with gastric perforation (7, 8), when the volume of effusion is small and when prior diagnostic or therapeutic interventions reduce bacterial counts (1). In such patients, laboratory biomarkers specific to sepsis that can be rapidly measured might offer high diagnostic accuracy and be faster than bacterial culture techniques (9, 10).

Studies in human and veterinary medicine have evaluated the utility of blood biomarkers for sepsis diagnosis (11–15). In people, effusion cytokine concentrations have been evaluated for identification of SP following gastrointestinal surgery and in particular colorectal surgery (16, 17), and may enable early identification of anastomotic leakage. The utility of cytokines for diagnosis of SP (either pre-operatively or post-operatively) has not been assessed in dogs to date, however.

The diagnostic and prognostic potential of novel blood biomarkers including cfDNA, nucleosomes, PCT and inflammatory cytokines have recently been investigated in dogs with sepsis (18–21). Procalcitonin is the precursor of the hormone calcitonin. In people with sepsis, PCT concentrations increase within 24 h in response to an infectious stimulus and likely originate from monocytes, neutrophils and liver, kidney, spleen, and lung (22). Functionally, PCT may modulate cytokines (23), affect vascular tone (24), and amplify inflammation (25). Studies of people with cirrhosis suggest that serum PCT measurement might aid in the identification of SP (26), although measurement in the ascitic fluid was not discriminating (27). Concentrations of PCT are increased in dogs with sepsis (20) and may have prognostic value (21).

Cell-free DNA is released from a variety of cell types through the processes of apoptosis, necrosis and the release of neutrophil extracellular traps (NETs) (28–30). This cfDNA can be present alone or in combination with various nuclear, cytoplasmic or granule proteins such as neutrophil elastase, cathepsin G, or myeloperoxidase. Nucleosomes are structures comprised of cfDNA and nuclear histone proteins (31). Although nucleosomes and cfDNA share common structural elements, they are distinct entities (32), with variable potential for immune cell activation through pattern recognition receptors (PRRs) (33). Measuring both biomarkers may provide better insights into the disease process than either alone (32). Immune cell recognition of molecular patterns like histones, nucleosomes and cfDNA by PRRs leads to production of cytokines (34), which may also aid identification of SP.

Cytokines are a very large and diverse group of signaling proteins and inflammatory mediators that includes chemokines, interleukins, interferons and growth factors. Their biology is complex because many of these factors are pleiotropic and their effect can vary with context, concentration and location (35). Interleukin-6 drives the hepatic acute phase protein response, promotes monocyte and B-cell differentiation and activates T cells (36). The cytokine CCL2 (previously known as MCP-1) is a chemokine that is primarily responsible for inducing and regulating the migration and infiltration of macrophages to the sites of inflammation produced by either tissue injury or infection. In mouse peritonitis models, CCL2 is the most important mediator of monocyte migration into the abdomen (37). Although it is produced by various cell types including epithelia, the major source of CCL2 is actually other macrophages (38).

Blood to fluid gradients for lactate and glucose have been reported to have high sensitivity and specificity for SP diagnosis in dogs (39, 40). However, confidence in these findings has been reduced by additional data (40, 41) that demonstrate similar gradients in effusions associated with non-septic ascites (NSA) due to cancer, pancreatitis, uroperitoneum and bile leakage (40, 42, 43). Clinically, differentiation of dogs with SP from those with NSA can be challenging because these patients often present with similar histories and comparable clinical signs. In some situations, the peritoneal effusions generated by these two syndromes may also be difficult to distinguish because inflammation of the peritoneum can arise secondary to a variety of stimuli (44), including bacteria (45) and biochemical irritants such as pancreatic secretions (46), bile (47), or urine (48).

The present study aimed to determine which blood and effusion biomarkers, and which blood-effusion biomarker concentration gradients were most able to discriminate SP from NSA in dogs. It was hypothesized that dogs with SP have blood and effusion biomarker concentrations greater than those in dogs with NSA, with the exception of glucose for which the blood and effusion concentrations were hypothesized to be smaller in dogs with SP than with NSA. It was hypothesized that dogs with SP have smaller blood-effusion biomarker gradients than in dogs with NSA, again with the exception of glucose for which the gradient was hypothesized to be greater in dogs with SP than with NSA.

## Materials and Methods

### Sample Size

Calculations were performed with an online calculator (http://www.quantitativeskills.com/sisa/), using pilot data from 15 dogs (5 SP, 10 NSA), with alpha set at 0.05 and beta set at 0.2. In the pilot data the mean ± SD cfDNA concentration in the peritoneal effusion of dogs with SP was 24,380 ± 8,356 pg/mL. The maximum value in the dogs with NSA was 15,800 pg/mL. To detect an equivalent difference (24,380–15,800 = 8,580 pg/mL) between two new populations of dogs where the overall SD was 8,356 pg/mL would have required 16 dogs per group.

### Animals

Dogs with evidence of systemic inflammatory response syndrome (SIRS) and a peritoneal effusion that were assessed in the emergency room and intensive care unit at the institution hospital between 08/16 and 03/18 were eligible for inclusion. The study was conducted with approval from the local Institutional Animal Care and Use Committee with informed client consent. Dogs were eligible for inclusion if they satisfied at least 2 of 4 SIRS criteria (49), and had peritoneal effusion identified using point-of-care ultrasonography performed according to established protocols (50, 51). The SIRS criteria used were temperature <100.6°F or >102.6°F; heart rate ≥120 beats per minute; respiratory rate ≥40 breaths per minute; white blood cell count >16,000 cells/μL or <6,000 cells/μL, or >3% band neutrophils. Dogs were categorized as having SP or NSA. The diagnosis of SP was based on positive bacterial culture or cytologic evidence of intracellular bacteria following evaluation of peritoneal effusion, or surgical confirmation of perforation of the gastrointestinal tract. The diagnosis of NSA was based on satisfaction of three criteria: (i) negative bacterial culture of peritoneal fluid, (ii) absence of cytologic evidence of bacteria, and (iii) establishment of an alternative final diagnosis (39). To minimize risks associated with blood or effusion sampling, dogs were excluded if they weighed <5 kg, had prothrombin time (PT) or activated partial thromboplastin time (aPTT) >150% upper reference interval value, or platelet count <30,000/μL.

### Data Collection

The following data were recorded at the time of sample collection: signalment, previous medical history, physical examination findings, non-invasive blood pressure, peripheral oxygen saturation, modified Glasgow coma scale score (52), and ultrasound body cavity fluid scores. This information enabled calculation of the shortened Acute Patient Physiologic and Laboratory Evaluation (APPLE_fast_) score (53). At the time of peritoneal fluid detection, blood samples and peritoneal fluid samples were obtained within approximately 10 minutes of each other, while ensuring no therapies were administered within this time period in order not to interfere with biomarker gradient estimations. None of the patients received blood products, colloids or vasopressors prior to sample collection. Peritoneal effusion samples were collected by percutaneous paracentesis and the fluid decanted into evacuated tubes containing lithium heparin, K2-EDTA, 3.2% sodium citrate or no additives. Infusion of fluid into the peritoneal cavity to facilitate recovery of effusion (i.e., diagnostic peritoneal lavage) was not performed for any patient in this study. Blood samples were collected from intravenous catheters or by venipuncture directly into evacuated tubes containing lithium heparin, K_2_-EDTA, 3.2% sodium citrate or no additives. Blood gas and electrolyte (RapidPoint 405, Siemens Medical Solutions, Norwood, MA), lactate (Lactate Pro, Arkray, Edina, MN) and refractometric total protein measurements were performed on heparinized blood and effusion samples immediately after collection. Serum and EDTA anticoagulated blood were submitted for routine chemistry testing (Cobas, Roche, Indianapolis, IN), and complete blood counts (ADVIA 2120, Siemens, Washington, DC). The remaining blood and peritoneal fluid samples were centrifuged for 10 min at 1370 rcf. The supernatants were separated and stored at −80°C pending batch analysis. Samples were analyzed in two batches to minimize storage duration. The maximum storage time between sampling and analysis was 12 months (median 3.5, IQR 2.5–5.3). On the day of batch analysis, samples were thawed and held at 4°C.

### Biomarker Assays

All biomarkers were measured in citrate-anticoagulated plasma and citrate-anticoagulated effusion samples. The present study used reagents and assays that have been previously validated (20, 54–62), or used by multiple independent groups for measurement of biomarker concentrations in dogs (18, 21, 63–68). Concentrations of cfDNA were measured using a benchtop fluorimeter (Qubit 3.0 Fluorometer) and relevant reagents (Quant-It HS dsDNA Kit), according to the manufacturers' instructions (Life Technologies, Waltham, MA). Samples were run in triplicate, and mean values used for statistical analysis, as previously reported (18, 68). Concentrations of plasma nucleosomes were analyzed using a commercial ELISA (Cell Death Detection ELISA Plus, Roche, Indianapolis, IN) scaled against pooled normal canine plasma because the assay lacks a reference standard (18, 68, 69). Plasma pooled from multiple normal dogs was analyzed in four wells on each plate and a reference value (assigned an arbitrary unit value of 1.0) was established as the mean of these four replicates. Nucleosome concentrations were expressed relative to the reference value (18, 68, 70). Concentrations of PCT were determined using a commercial kit (Biovendor, Asheville, NC), as previously described (20, 21). A previously validated kit (Biomedica Gruppe, Vienna, Austria), was used to measure NT-proCNP concentrations (57, 62). An antibody-coated microsphere-based multiplex cytokine immunoassay kit (CCYTOMAG-90K, EMD-Millipore, Billerica, MA), designed for the simultaneous quantification of multiple cytokines was used as previously described (64, 71). This assay was performed with overnight sample incubation at 4°C to maximize sensitivity. Analytes included were CCL2, CXCL8, IL-6, IL-10, and KC-Like. Cytokine concentrations were measured in duplicate and the mean values used for subsequent analyses. The observed concentration of each analyte for each sample was calculated using a standard curve generated from the standards and blank provided by the manufacturer [the median (min-max) *r*^2^ values for standard curves was 1.000 (0.996–1.000)]. Where values were recorded as below the measurable range of the assay the manufacturer's stated minimum detectable concentration was imputed as follows: 21.0 pg/mL (CCL2), 21.7 pg/mL (CXCL8), 20 mg/dL (glucose), 3.7 pg/mL (IL-6), 8.5 pg/mL (IL-10), and 5.3 pg/mL (KC-like).

### Statistical Analyses

Prior to test selection, data were assessed for normality using the D'Agostino Pearson test and descriptive statistics calculated accordingly. The majority of variables were not normally distributed. In addition, cytokine concentrations were assumed to be non-parametric due to imputation of data for the lower limit of detection for some measurements. All data are therefore presented as median (interquartile range) and non-parametric hypothesis tests were used throughout. Case fatality rates were compared between the two groups using Fisher's exact test. Biomarker concentrations were compared between pairs of blood and effusion samples within the SP group and within the NSA group with the Wilcoxon matched-pairs signed rank test. Between dogs with SP and with NSA, biomarker concentrations in blood, in effusion and the blood-effusion gradients were compared using the Mann-Whitney *U*-test. Alpha was set at 0.05 for all hypothesis tests, with *post-hoc* Bonferroni corrections applied to adjust for multiple comparisons. Box and whisker plots were also generated to visualize comparisons.

The discriminating ability of biomarkers for the diagnosis of SP was evaluated by plotting receiver-operating characteristic (ROC) curves and through calculation of the area-under these curves (AUROC). Specifically, blood concentrations, effusion concentrations and the blood-effusion gradients of each biomarker were evaluated separately. Optimal cutoffs for sensitivity and specificity were identified by maximizing the Youden index = (Sensitivity+Specificity)-1 (72). Values for AUROC between models were compared per Hanley and McNeil (73, 74). Analyses were performed using commercial software (Prism 6.0, GraphPad, La Jolla, CA; SPSS Statistics 24, IBM, Armonk, NY).

## Results

### Study Population Characteristics

A total of 37 dogs were enrolled, 18 with SP and 19 with NSA. The SP group included mixed breed dogs (*n* = 4), Labrador retriever, beagle hound, and Siberian husky (all *n* = 2), and 1 each of the following: shih tzu, dachshund, American Eskimo, mastiff, bichon frise, golden retriever, rottweiler, and border collie. The NSA group included Labrador retriever (*n* = 4), border collie (*n* = 3), mixed breed (*n* = 3), and 1 each of the following: Australian shepherd, German short haired pointer, pit bull terrier, Bernese mountain dog, Havanese, foxhound, soft-coated wheaten terrier, golden retriever and Australian cattle dog. The SP group included 10 female spayed and 8 male castrated dogs, while the NSA group included 9 female spayed and 10 male castrated dogs. The median age in the SP group was 8 (4.8–10.3) years, and in the NSA group 9 (6–10) (*P* = 0.510). The median APPLE_fast_ score in the SP group was 22 (IQR 20-29) and in the NSA group 21 (IQR 16-24) (*P* = 0.143). Median bodyweight in the SP group was 26 (13–35) kg and in the NSA group 32 (21–41) kg (*P* = 0.218) (Supplementary Table 1). Of the SP group, 8/18 cases were primary emergencies (10/18 were referred) and in the NSA group 3/19 cases were primary emergencies (16/19 were referred). Of the 18 SP dogs, 10 were euthanized perioperatively due to poor or worsening prognosis, 1 experienced cardiopulmonary arrest and died, and 7 were discharged alive (61.1% fatality). Of the 19 NSA dogs, 8 were euthanized due to poor or worsening prognosis and 11 were discharged alive. Of the 11 dogs discharged alive, however, 1 was discharged against medical advice and another to be euthanized at home (44.4% fatality with 1 censored case). There was no difference in case fatality rates between the two groups (*P* = 0.505). Causes of SP included intestinal perforation due to neoplasia (*n* = 3), foreign body (*n* = 2), gallbladder rupture with septic bile peritonitis (*n* = 3), dehiscence of a previous gastrointestinal surgical site (*n* = 2), liver abscess (*n* = 1), pancreatic abscess (*n* = 1), mesenteric lymph node abscess (*n* = 1), septic uroperitoneum from urinary bladder rupture (*n* = 1), and unknown source (*n* = 4). The four dogs with SP of unknown source were euthanized following cytologic diagnosis of septic peritoneal effusion but prior to further investigation of the origin of the SP. The diagnosis of SP was based on positive bacterial culture or cytologic evidence of intracellular bacteria following evaluation of peritoneal effusion (*n* = 15, 83%), or surgical confirmation of perforation of the gastrointestinal tract (*n* = 3, 17%) (2). Causes of NSA included sterile uroperitoneum (*n* = 4), bile peritonitis (*n* = 3), urethral obstruction (*n* = 2), recent laparotomy for gastrointestinal surgery (*n* = 2), pancreatitis (*n* = 2), gastric dilatation and volvulus (*n* = 1), neoplasia of hematopoietic origin (*n* = 1), acute renal failure (*n* = 1), intestinal foreign body (*n* = 1), intestinal adhesions (*n* = 1), and lymphoma (*n* = 1).

### Biomarker Concentrations in Blood and Peritoneal Effusion

To determine if biomarker concentrations were different between peripheral blood and peritoneal effusion, pairwise comparisons between blood and effusion biomarker concentrations were performed. These results are summarized in Table 1. In dogs with SP, the concentrations of glucose, cfDNA, nucleosomes, PCT, CCL2, IL-6, IL-10, and KC-like were significantly different between blood and peritoneal effusion. For all biomarkers except glucose and PCT, the concentrations of these analytes were greater in the effusion compared to the blood of dogs with SP. In contrast, in dogs with NSA, only the concentrations of CCL2, IL-6 and KC-like were different in the effusion compared to blood; here also the effusion concentrations were greater than those in blood.

**Table 1:** Comparisons of blood biomarker concentration with effusion biomarker concentrations in dogs with septic peritonitis (SP) and in dogs with non-septic peritonitis (NSA).

	**SP**				**NSA**			
	**Blood median IQR**	**Effusion median IQR**	***P*-value**	***P*_**corr**_**	**Blood median IQR**	**Effusion median IQR**	***P*-value**	***P*_**corr**_**
Glucose (mg/dL)	111	20	0.0011	**0.0121**	101	94	0.1648	1.8128
	81–124	20–108			79–118	78–99		
Glucose (mmol/L)	6.2	1.1	0.0011	**0.0121**	5.6	5.2	0.1648	1.8128
	4.5–6.9	1.1–6.0			4.4–6.6	4.4–5.5	0.1648	1.8128
Lactate (mmol/L)	2.7	7.9	0.0066	0.0726	2.0	1.8	0.6998	7.6978
	1.2–4.2	2.7–11.3			1.3–2.6	1.1–2.8		
cfDNA (ng/mL)	379	1085	0.0003	**0.0033**	410	470	0.7381	8.1191
	316–430	493–2723			316–629	269–1157		
Nucleosomes (AU)	0.35	13.1	0.0001	**0.0011**	0.6	2.5	0.0289	0.1848
	0.2–1.1	4.4–25.1			0.3–1.8	0.3–12.9		
NT–proCNP (pmol/L)	11.9	6.2	0.2460	2.910	3.9	2.3	0.1134	1.333
	5.6–42.1	1.9–25.7			2.3–12.0	1.6–10.3		
PCT (pg/mL)	99.6	38.4	0.0028	**0.0352**	93.5	98.9	0.6226	6.8486
	40.3–182.9	0.0–119.2			54.7–208.1	29.7–201.6		
CCL2 (pg/mL)	1274	16066	0.0001	**0.0011**	249	683	0.0001	**0.0011**
	282–2715	2508–96300			21–431	449–2225		
CXCL8 (pg/mL)	785.4	4325.0	0.0090	0.099	550.3	129.6	0.1901	2.0911
	495.6–1857.0	21.7–75000.0			51.8–1372.0	21.7–444.7		
IL-10 (pg/mL)	8.5	303.1	0.0012	**0.0132**	8.5	8.5	0.0312	0.3432
	8.5–106.4	147.6–1584.0			8.5–8.5	8.5–31.9		
IL-6 (pg/mL)	9.9	2775.0	0.0001	**0.0011**	3.7	37.9	0.0029	**0.0319**
	3.7–1573.0	363.5–11495.0			3.7–15.9	3.7–1918.0		
KC-Like (pg/mL)	164.1	1215.0	0.0011	**0.0121**	20.3	456.2	0.0001	**0.0011**
	8.0–1137.0	37.2–6973.0			5.3–71.5	62.1–757.0		

*Median and IQR concentrations of measured biomarkers in blood and effusion in patients with SP and NSA, and results of univariate comparisons of biomarker concentrations between fluid types with each group by Wilcoxon matched-pairs signed rank test. Biomarkers significantly different after correction for multiple comparisons (P <0.05) between blood and effusion within SP and NSA groups are indicated in bold typeface. AU, arbitrary unit; CCL2, C-C Motif Chemokine Ligand-2; cfDNA, cell-free DNA; CXCL8, chemokine C-X-C motif ligand 8; IL, interleukin; IQR, interquartile range; KC-Like, keratinocyte chemoattractant-like; NSA, non-septic ascites; NT-proCNP, amino-terminal propeptide of C-type natriuretic peptide; P_corr_, P-value corrected for multiple comparisons; PCT, procalcitonin; SP, septic peritonitis*.

### Comparisons of Biomarker Concentrations in SP and NSA

**Figure 1:**
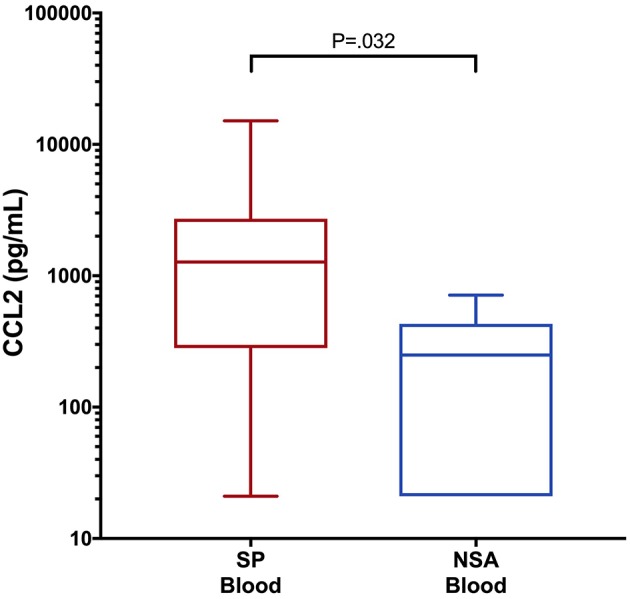
Box and whisker plot of blood CCL2 concentrations in dogs with SP and NSA. The central line within the box indicates the median, the box represents the interquartile range and the whiskers represent the minimum and maximum values. *P* <0.05 was after correction for multiple comparisons was considered significant. CCL2, C-C Motif Chemokine Ligand-2; IL, interleukin.

**Figure 2:**
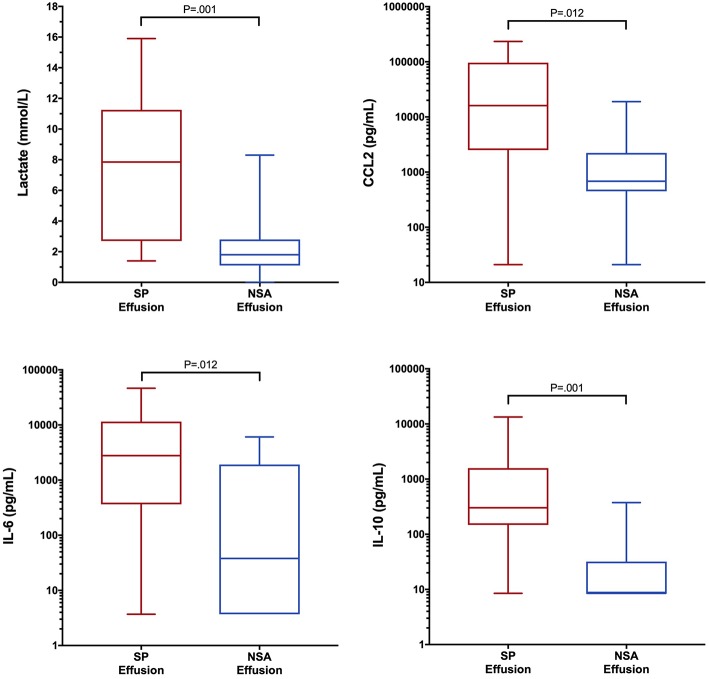
Box and whisker plot of effusion lactate, CCL2, IL-6 and IL-10 concentrations in dogs with SP and NSA. The central line within the box indicates the median, the box represents the interquartile range and the whiskers represent the minimum and maximum values. *P* <0.05 was after correction for multiple comparisons was considered significant. CCL2, C-C Motif Chemokine Ligand-2; IL, interleukin.

**Figure 3:**
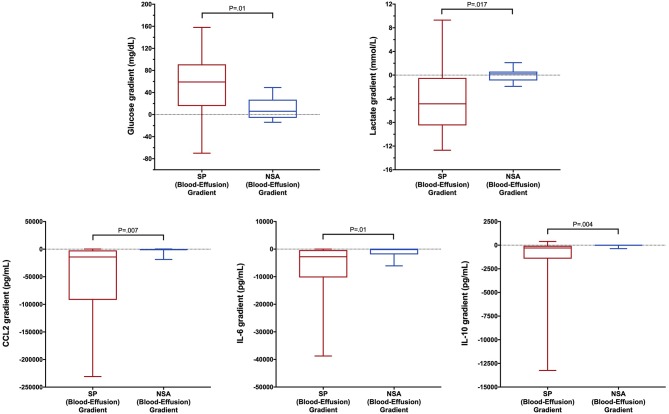
Box and whisker plot of blood-effusion gradients of glucose, lactate, CCL2, IL-6, and IL-10 concentrations in dogs with SP and NSA. The central line within the box indicates the median, the box represents the interquartile range and the whiskers represent the minimum and maximum values. *P* <0.05 was after correction for multiple comparisons was considered significant. CCL2, C-C Motif Chemokine Ligand-2; IL, interleukin.

To determine if blood biomarker concentrations could discriminate dogs with SP from those with NSA, the concentrations of each of the blood biomarkers were compared between the two groups of dogs. These data are summarized in Tables 2–4. After correction for multiple within group comparisons, only CCL2 was significantly different between dogs with SP and those with NSA. Specifically, blood CCL2 was higher in dogs with SP than with NSA (Figure 1). Similarly, to determine if effusion biomarker concentrations could discriminate SP from NSA, the concentration of the effusion biomarkers were compared between the two groups of dogs. Here, the effusion concentrations of lactate, CCL2, IL-6, and IL-10 were significantly greater in dogs with SP compared to those with NSA after correction for multiple comparisons (Figure 2). Finally, the blood-effusion concentration gradients were compared between dogs with SP and dogs with NSA. There were significantly smaller blood-effusion gradients of lactate, CCL2, IL-6, and IL-10 in dogs with SP compared to NSA (Figure 3). There was a significantly larger blood-effusion gradient of glucose in dogs with SP compared to dogs with NSA.

**Table 2:** Comparisons of blood biomarker concentrations in dogs with septic peritonitis (SP) and non-septic peritonitis (NSA).

	**Blood**			
	**SP median IQR**	**NSA median IQR**	***P*-value**	***P*_**corr**_**
Glucose (mg/dL)	111	101	0.7927	8.7197
	81–124	79–118		
Glucose (mmol/L)	6.2	5.6	0.7927	8.7197
	4.5–6.9	4.4–6.6		
Lactate (mmol/L)	2.7	2.0	0.3698	4.0678
	1.2–4.2	1.3–2.6		
cfDNA (ng/mL)	379	410	0.3953	4.3483
	316–430	316–629		
Nucleosomes (AU)	0.35	0.60	0.2043	2.2473
	0.2–1.1	0.3–1.8		
NT-proCNP (pmol/L)	11.9	3.9	0.0399	0.4389
	5.6–42.1	2.3–12.0		
PCT (pg/mL)	99.6	93.5	0.7299	8.0289
	40.3–182.9	54.7–208.1		
CCL2 (pg/mL)	1274	249	0.0029	**0.0319**
	282–2715	21–431		
CXCL8 (pg/mL)	785.4	550.3	0.188	2.068
	495.6–1857.0	51.8–1372.0		
IL-10 (pg/mL)	8.5	8.5	0.008	0.088
	8.5–106.4	8.5–8.5		
IL-6 (pg/mL)	9.9	3.7	0.0680	0.748
	3.7–1573.0	3.7–15.9		
KC-Like (pg/mL)	164.1	20.3	0.0731	0.8041
	8.0–1137.0	5.3–71.5		

*Median and IQR concentrations of measured biomarkers in blood in patients with SP and NSA, and results of univariate comparisons of biomarker concentrations between groups by Mann-Whitney U-test. Biomarkers identified to be significantly different in blood compared between SP and NSA groups after correction for multiple comparisons (P <0.05) are indicated in bold typeface. AU, arbitrary unit; CCL2, C-C Motif Chemokine Ligand-2; cfDNA, cell-free DNA; CXCL8, chemokine C-X-C motif ligand 8; IL, interleukin; IQR, interquartile range; KC-Like, keratinocyte chemoattractant-like; NSA, non-septic ascites; NT-proCNP, amino-terminal propeptide of C-type natriuretic peptide; P_corr_, P value corrected for multiple comparisons; PCT, procalcitonin; SP, septic peritonitis*.

**Table 3:** Comparisons of effusion biomarker concentrations in dogs with septic peritonitis (SP) and non-septic peritonitis (NSA).

	**Effusion**			
	**SP median IQR**	**NSA median IQR**	***P*-value**	***P*_**corr**_**
Glucose (mg/dL)	20	94	0.011	0.121
	20–108	78–99		
Glucose (mmol/L)	1.1	5.2	0.011	0.121
	1.1–6.0	4.3–5.5		
Lactate (mmol/L)	7.9	1.8	0.0001	**0.0011**
	2.7–11.3	1.1–2.8		
cfDNA (ng/mL)	1085	470	0.0205	0.2255
	493–2723	269–1157		
Nucleosomes (AU)	13.1	2.5	0.0056	0.0616
	4.4–25.1	0.3–12.9		
NT-proCNP (pmol/L)	6.2	2.3	0.2806	3.0866
	1.9–25.7	1.6–10.3		
PCT (pg/mL)	38.4	98.9	0.0489	0.5379
	0–119.2	29.7–201.6		
CCL2 (pg/mL)	16066	683	0.0011	**0.0121**
	2508–96300	449–2225		
CXCL8 (pg/mL)	4325.0	129.6	0.0113	0.1243
	21.7–75000.0	21.7–444.7		
IL-10 (pg/mL)	303.1	8.5	0.0001	**0.0011**
	147.6–1584.0	8.5–31.9		
IL-6 (pg/mL)	2775.0	37.9	0.0011	**0.0121**
	363.5–11495.0	3.7–1918.0		
KC-Like (pg/mL)	1215.0	456.2	0.312	3.432
	37.2–6973.0	62.1–757.0		

*Median and IQR concentrations of measured biomarkers in effusion in patients with SP and NSA, and results of univariate comparisons of biomarker concentrations between groups by Mann-Whitney U-test. Biomarkers identified to be significantly different in effusion compared between SP and NSA groups after correction for multiple comparisons (P <0.05) are indicated in bold typeface. AU, arbitrary unit; CCL2, C-C Motif Chemokine Ligand-2; cfDNA, cell-free DNA; CXCL8, chemokine C-X-C motif ligand 8; IL, interleukin; IQR, interquartile range; KC-Like, keratinocyte chemoattractant-like; NSA, non-septic ascites; NT-proCNP, amino-terminal propeptide of C-type natriuretic peptide; P_corr_, P value corrected for multiple comparisons; PCT, procalcitonin; SP, septic peritonitis*.

**Table 4:** Comparisons of blood-effusion biomarker concentration gradients in dogs with septic peritonitis (SP) and non-septic peritonitis (NSA).

	**Blood-Effusion Gradient**			
	**SP median IQR**	**NSA median IQR**	***P*-value**	***P*_**corr**_**
Glucose (mg/dL)	59	6	0.0009	**0.0099**
	16–91	−6–27		
Glucose (mmol/L)	3.3	0.3	0.0009	**0.0099**
	0.9–5.1	−0.3–1.5		
Lactate (mmol/L)	−4.9	0.2	0.0015	**0.0165**
	−8.5 to −0.5	−0.9–0.6		
cfDNA (ng/mL)	−757	−31	0.005	0.055
	−2331 to −179	−527–165		
Nucleosomes (AU)	−13.0	−0.7	0.0066	0.0726
	−22.5 to −3.4	−12.8–0.3		
NT-proCNP (pmol/L)	2.4	0.8	0.3744	4.1624
	−1.6–7.4	−0.5–2.6		
PCT (pg/mL)	27.9	22.4	0.0861	0.9471
	8.3–98.7	−32.7–33.7		
CCL2 (pg/mL)	−14151	−561	0.0006	**0.0066**
	−91817 to −2243	−1827 to −110		
CXCL8 (pg/mL)	−3657.0	134.1	0.0129	0.1419
	−68631.0–623.7	−51.1–1316.0		
IL-10 (pg/mL)	−294.6	0.0	0.0004	**0.0044**
	−1430.0 to −63.8	−23.4 to 0.0		
IL-6 (pg/mL)	−2772.0	−0.5	0.0009	**0.0099**
	−10272.0 to −359.8	−1914.0 to 0.0		
KC-Like (pg/mL)	−937.3	−425.5	0.3786	4.1646
	−4615.0 to −31.9	−716.7 to −41.3		

*Median and IQR blood-effusion concentration gradients of measured biomarkers in patients with SP and NSA, and results of univariate comparisons of biomarker concentration gradients between groups by Mann-Whitney U-test. Biomarker bloodeffusion gradients identified to be significantly different compared between SP and NSA groups after correction for multiple comparisons (P <0.05) are indicated in bold typeface. AU, arbitrary unit; CCL2, C-C Motif Chemokine Ligand-2; cfDNA, cell-free DNA; CXCL8, chemokine C-X-C motif ligand 8; IL, interleukin; IQR, interquartile range; KC-Like, keratinocyte chemoattractant-like; NSA, non-septic ascites; NT-proCNP, amino-terminal propeptide of C-type natriuretic peptide; P_corr_, P value corrected formultiple comparisons; PCT, procalcitonin; SP, septic peritonitis*.

### Predicting Septic Peritonitis

**Figure 4:**
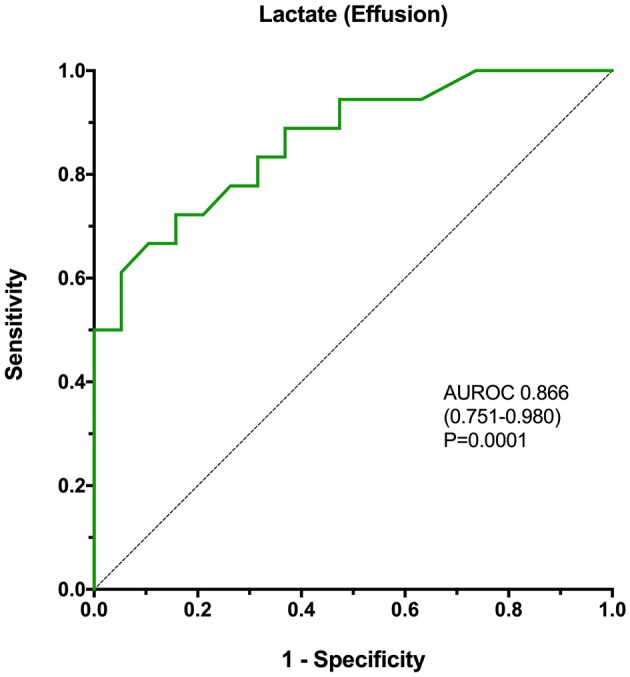
A receiver operating characteristic curve analysis of the ability of effusion lactate concentration to discriminate SP from NSA in dogs, with calculation of the area under the curve, associated confidence intervals and *P*-value. The *P*-value represents the significance of the comparison of the area under the curve with the line of identity (AUROC = 0.5). AUROC, area under the receiver operating characteristic curve.

The comparisons between blood and effusion biomarker concentrations described above were used to identify candidates for ROC curve generation. Specifically, ROC curves were generated for blood concentrations of CCL2, for effusion concentrations of lactate, CCL2, IL-6, and IL-10 and for blood-effusion gradients of lactate, glucose, CCL2, IL-6, and IL-10. The AUROC values with 95% confidence intervals (CI) and associated *P*-values are presented in Table 5. Based on these AUROC values, the most discriminant biomarker was the effusion lactate concentration (Figure 4), AUROC 0.866, 95% CI 0.751–0.980 (*P* = 0.0001). None of the AUROC values in Table 5 were significantly different from each other, however. Calculation of the Youden index identified that a cut-off for the effusion lactate concentration of 4.2 mmol/L was 72.2% sensitive (95% CI 46.5–90.3) and 84.2% specific (95% CI 60.4–96.6) for SP. The Youden index identified that a cut-off for the blood-effusion glucose gradient of <37 mg/dL (<2.06 mmol/L) was 89.5% sensitive (95% CI 66.9–98.7) and 66.7% specific (95% CI 41.0–86.7).

**Table 5:** Biomarker area under the receiver operating characteristic curve values for the discrimination of septic peritonitis from non-septic ascites in dogs.

**Parameter**	**AUROC**	**95% Confidence interval**	***P*-value**
CCL2 (Blood)	0.778	0.625–0.931	0.0039
Lactate (Effusion)	0.866	0.751–0.980	0.0001
CCL2 (Effusion)	0.804	0.647–0.961	0.0016
IL-6 (Effusion)	0.803	0.662–0.943	0.0017
IL-10 (Effusion)	0.836	0.694–0.978	0.0005
Lactate (Gradient)	0.797	0.630–0.963	0.0020
Glucose (Gradient)	0.809	0.653–0.964	0.0013
CCL2 (Gradient)	0.817	0.667–0.968	0.0010
IL-6 (Gradient)	0.807	0.669–0.946	0.0014
IL-10 (Gradient)	0.817	0.662–0.973	0.0010

*Area under the receiver operating characteristic curve values for the ability of measured biomarkers to discriminate dogs with SP and NSA. P-values represent the significance of the difference between the AUROC value and 0.5. AUROC, area under the receiver operating characteristic curve; CCL2, C-C Motif Chemokine Ligand-2; IL, interleukin*.

## Discussion

Differentiating SP from NSA can be challenging as many of these patients have similar presenting complaints, clinical examination abnormalities, and clinicopathological findings. Early diagnosis of SP might improve outcomes through expedited administration of antimicrobials and definitive surgical correction (75, 76). Definitively identifying NSA might prevent unnecessary antimicrobial drug administration and surgical exploration of dogs with NSA, reducing morbidity, client cost and patient risk. As such, finding a way to reliably, rapidly and non-invasively segregate these two populations would greatly aid clinicians. Previous studies have focused primarily on measurements of blood biomarker concentrations (12, 13, 18, 19, 57, 59), while only one study evaluated NT-proCNP measured in both blood and effusion for the diagnosis of SP (62). The present study aimed to address this unmet need by evaluating the ability of various biomarkers, measured both in blood and in effusion, to distinguish between SP and NSA.

In the present study, the marker that best differentiated SP from NSA was the effusion lactate concentration. This result is consistent with previous findings, although the discriminating ability in the present study was comparatively diminished (39, 40). In the study by Levin et al. involving 8 dogs with SP, a fluid lactate concentration above 2.5 mmol/L had a 100% sensitivity and 91% specificity for diagnosing SP (40). The study by Bonczynski et al. involving 7 dogs with SP did not indicate the diagnostic accuracy of the effusion lactate concentration alone; however, that study reported that a blood-effusion lactate gradient greater than −2mmol/L was 100% sensitive and 100% specific for the diagnosis of SP in dogs (39). We speculate that the differences between those findings and ours are due to the nature of the control population in the present study. We specifically selected dogs with NSA that clinically were very similar to those with SP. Specifically the two populations had comparable clinical histories, similar physical examination findings and illness severity scores. This likely resulted in a control population of dogs with inflammatory peritoneal effusions that were less easily differentiated from dogs with SP as compared to the control populations in earlier publications.

The lactate measured in the peritoneal effusion of dogs with SP may originate from bacterial metabolism, from the activity of infiltrating leukocytes or from diffusion from blood (40). The present study suggests it may be a combination of all these factors. If the lactate in septic effusions resulted from bacterial metabolism alone, then the lactate concentrations in non-septic effusions should be very low, or equal to blood concentrations due to diffusion. In the present study, effusion lactate concentrations were significantly greater in SP than in NSA, but some overlap existed (see Figure 2), indicating a source of lactate other than bacterial metabolism was likely present. Point of care lactate meters presently measure only L-lactate and cannot identify the D-lactate isoform predominantly produced by bacterial metabolism. Improved discrimination of the two patient populations evaluated here might be possible with measurement of both D- and L-lactate. We speculate that the infiltrating leukocytes may also have been generating lactate through altered cellular metabolism (77) in an environment with low oxygen tension (78).

The blood-effusion glucose gradient was significantly higher in the dogs with SP as compared to dogs with NSA, a finding that is consistent with a previous study (39). The alterations in glucose concentrations may reflect glucose consumption by bacterial and leukocyte metabolism. Dogs with SP had significantly greater effusion concentrations of CCL2, IL-6, and IL-10 compared to dogs with NSA. In addition, blood-effusion gradients of these cytokines were also significantly smaller in dogs with SP compared to those with NSA. These findings suggest effusion concentrations of CCL2, IL-6, and IL-10 may be more specific to bacterial infection rather than reflecting only the presence of an inflammatory process. Two of these cytokines are pro-inflammatory (IL-6 and CCL2), while IL-10 is anti-inflammatory. We speculate that the difference in the concentrations of this cytokine between SP and NSA patients may reflect greater levels of macrophage activity in the peritoneal cavities of dogs with SP compared to dogs with NSA. Future investigations might assess macrophage counts in SP and NSA patients to evaluate this hypothesis. Interleukin-10 is involved in down-regulating macrophage and helper T-cell function and is a key immunoregulator during infection that may mitigate tissue damage from excessive cytotoxicity. It has been demonstrated that IL-10 concentrations correlate with pathogen burden in some disease processes (79), which may in part explain its discriminating ability in the present study.

The only significant difference in blood biomarker concentrations was for CCL2, which was significantly greater in dogs with SP compared to NSA. In canine studies modeling sepsis through injection of lipopolysaccharide (LPS), treated dogs had greater blood IL-6, IL-10, TNF-α, and KC-like concentrations up to 4-h post-injection and greater CCL2 up to 24-h post-injection, when compared to placebo-treated dogs (34). Other studies have demonstrated that CCL2 may be prognostic in dogs with IMHA (71), lymphoma (80), and babesiosis (81).

In this study, the predominant sources of infection in dogs with SP were the gastrointestinal and biliary systems, findings that are consistent with previous reports (3, 82, 83). The predominant sources of effusion in dogs with NSA were the urinary tract and the biliary system. Both bile peritonitis and uroperitoneum can lead to sterile or septic abdominal effusions. The presence of sepsis in addition to the chemical peritonitis that results from these conditions might influence treatment decisions and could potentially affect prognosis. Although bile peritonitis and uroperitoneum can be identified using measurements of bilirubin or potassium, these biochemical tests do not distinguish septic from non-septic effusions, which suggests there is still a need for reliable biomarkers to distinguish SP from NSA. The case fatality rate (including euthanasia) in this population of dogs with SP was 61.1%, consistent with previous findings (2, 4). The case fatality rate for dogs with NSP was 44.4%. While both populations had comparable APPLE_fast_ scores the overall case fatality rates are higher for both populations than would be expected based on the APPLE_fast_ scores alone. There are several potential explanations for this. Some patients in the SP group may have been euthanized for a perceived poor prognosis, yet might have survived with intensive care. Some of the patients in the NSA group may have been euthanized due to current illness secondary to an underlying terminal disease (e.g., cancer). Likewise, while most patients in the study were referred to us it is possible that financial considerations played a role in euthanasia decisions. It is also possible that differences between our institution and the center where the APPLE score was developed may also explain part of this discrepancy.

Previous studies in mouse models and in people suggest that in SP, inflammatory biomarker concentrations and markers of NET formation are greater in the peritoneal effusion than in blood, while glucose concentrations are lower (16, 17, 84–86). In dogs with SP in the present study, multiple biomarker concentrations were significantly different between effusion and blood including glucose, cfDNA, nucleosomes, PCT, CCL2, IL-6, IL-10, and KC-like. In dogs with NSA, only IL-6 and KC-like concentrations were different between blood and effusion. While the innate immune responses to pathogen- and damage-associated molecular patterns may be qualitatively similar (33, 87, 88), the response incited by bacteria may be more intense than that incited by tissue damage alone. It could also suggest that adaptive immune responses and collaborative interactions between leukocytes reacting to the presence of bacteria leads to a distinct, or a greater inflammatory response than tissue damage alone. The high concentrations of cfDNA and nucleosomes in the peritoneal effusions of SP dogs is also very suggestive that NET release was occurring in the peritoneal cavities of these dogs, as has been previously reported (89). The increased blood PCT concentrations relative to those in effusion identified in the present study is consistent with previous findings of high PCT concentrations in dogs with sepsis (20, 21). Comparably, a study in people with cirrhosis also identified increased serum PCT concentrations in those patients with SP (26). Our findings are also consistent with a subsequent study, also in cirrhotic people, which demonstrated that blood-effusion PCT gradients were not diagnostic of SP (27).

The present study is not without limitations. The required sample size for this study was estimated based on pilot data on cfDNA concentrations only. This may have increased the risk of a type II error in our comparisons of other biomarkers between SP and NSA populations. Our study population included both primary emergency and secondary referral cases, and thus some dogs in the present study received treatment from other veterinarians prior to referral and study enrollment. These therapies included intravenous fluid therapy and antimicrobials that might have altered biomarker concentrations. Given the variable nature of type and timing of these prior therapies, it is difficult to account for these interventions in the analyses of the biomarker concentrations and it remains possible that prior therapy could both falsely increase and falsely decrease biomarker concentrations. It is not known how rapidly blood and effusion concentrations change or equilibrate in response to such therapies, making it is difficult to assess the effect of prior interventions on our results. The most discriminating biomarker was effusion lactate concentration, which may have been less affected by therapies administered before study enrollment and sample collection. While gastrointestinal perforation typically leads to SP, there may be a delay between the occurrence of perforation and the characteristic inflammatory response due to the leakage of bacteria and ingesta into the abdomen. In cases where perforation has only very recently occurred the utility of biomarkers of the inflammatory response for the diagnosis of SP might be reduced. The timing of any suspected gastrointestinal perforation should be considered when evaluating the results of biomarker analyses.

Most blood samples were obtained from the cephalic vein at the time of catheter placement, but some samples were drawn by direct venipuncture or from central venous catheters. It is possible that these differences in sampling location might have influenced biomarker concentrations, in particular lactate. The effects of the variability on biomarker concentrations was likely small, but could have been sufficient to alter the calculated gradients. Uniform, consistent cytologic evaluation of peritoneal fluid samples was not performed as part of the present study. Evaluation of peritoneal fluid samples was typically performed by ER personnel with various levels of training, which could have led to false negatives in some patients with NSA.

The SIRS criteria were used to screen patients for this study because they provide rapid, simple and objective identification of the potential presence of systemic inflammation. In people it is recognized that these criteria are neither sensitive or specific for sepsis (90, 91). They should only be applied to patients with compatible history, clinical signs, and physical examination findings that raise concern for sepsis or another disease process likely to cause a severe physiologic stress response. If the SIRS criteria are applied without this context specificity then they cease to be informative. In human medicine sepsis was recently redefined using a data driven approach to enhance the specificity of the clinical criteria used to identify the syndrome. These Sepsis-3 definitions incorporate a rapid organ failure assessment score (92), that has yet to be robustly examined in veterinary medicine. An equivalent redefinition of sepsis in veterinary medicine had not been undertaken and hence the SIRS criteria remain the best available strategy (93), and have been used in multiple studies of canine sepsis and of septic peritonitis specifically (6, 14, 94, 95). As such, they were considered to be a necessary component of the identification of the SP and NSA populations in the present study. Use of the SIRS criteria facilitated identification of a control population of dogs with similar physical examination parameters but NSA rather than SP, such as dogs with pancreatitis that can be difficult to distinguish clinically from SP.

The biomarker assays used here have been validated or widely used to measure concentrations in blood samples in dogs, but only one of these assays has been specifically validated for use on effusion samples (62). Other groups have used the same assays to measure concentrations of markers including glucose, lactate, and NT-proCNP in both effusion and blood samples (39, 40, 62). In addition, multiple studies in humans have measured cytokines in peritoneal fluid samples using comparable assays to those used here (16, 96–100). The effusion samples we analyzed were the supernatants of the peritoneal fluid, centrifuged and separated prior to storage and hence are ultrafiltrates of plasma modified by local effects such as glucose consumption or lactate addition. Validation of the assays used on abdominal fluid samples was not performed in this study and represents a significant limitation to our data. We cannot exclude the possibility that matrix effects or the presence of interfering substances could have affected our results in unpredictable ways. Our data should be considered preliminary until such comprehensive validation work is conducted.

In the present study, the NSA group included a mixture of underlying disease processes with diverse pathophysiology. It is possible that the results of the present study might be different if a single cause of SP such as intestinal perforation had been compared with a single cause of NSA such as pancreatitis. Likewise, the biomarkers evaluated here might perform differently if direct comparisons of septic uroperitoneum or bile peritonitis vs. sterile uroperitoneum or sterile bile peritonitis had been conducted. The present study measured blood and effusion biomarker concentrations at a single time point only and likely at different time points within the disease process for different patients. Our conclusions about the diagnostic utility of these biomarkers are based on these single time point observations only. Biomarker concentrations change over the time course of disease and had measurements had been performed at other times distinct conclusions about the utility of specific biomarkers might have been reached. The present study enrolled client-owned dogs with naturally occurring disease and hence it was not feasible to standardize the timing of sample collection or to collect serial peritoneal fluid samples from patients with septic peritonitis. Our findings are also generalizable to other real-world settings where samples would likely be collected at the time of presentation or when the peritoneal fluid was first identified.

In conclusion, the results of this study support the current practice of measurement of effusion lactate concentrations for the discrimination of SP from NSA. It should be recognized that no single biomarker is completely discriminating for SP and hence no single concentration cut-off offers 100% sensitivity and specificity. Measurement of multiple biomarkers in blood and effusion might provide a more accurate identification of SP. Several novel biomarkers including pro- and anti-inflammatory cytokines and markers of NET formation may also be of value in distinguishing SP from NSA, but confirmation of those findings in other populations is warranted before they can be recommended. Future studies evaluating biomarker concentrations in blood or fluid for the diagnosis of sepsis will need to carefully select the control populations in order to adequately test the discriminating ability of the biomarker in real-world clinically relevant scenarios.

## Data Availability

The datasets generated for this study are available on request to the corresponding author.

## Ethics Statement

All samples analyzed in this study were collected from dogs managed at the participating veterinary teaching hospital as part of studies approved by the local Institutional Animal Care and Use Committees (IACUC), and undertaken under written informed client consent (Cornell IACUC 2014-0053).

## Author Contributions

PM assisted with study design, collected, and analyzed data and co-wrote the manuscript. RG designed the study, collected, and analyzed data and co-wrote the manuscript. Both authors contributed to, read and approved the final manuscript.

### Conflict of Interest Statement

The authors declare that the research was conducted in the absence of any commercial or financial relationships that could be construed as a potential conflict of interest.

## Supplementary Material

Supplementary Table 1 A summary of the demographics and physical examination characteristics of the two patient populations septic peritonitis (SP) and non-septic ascites (NSA).

## Abbreviations

APPLE: acute patient physiologic and laboratory evaluation
aPTT: activated partial thromboplastin time
AU: arbitrary unit
AUROC: area under the ROC curve
CCL2: C-C Motif Chemokine Ligand-2
cfDNA: cell-free DNA
CI: confidence interval
CXCL8: chemokine C-X-C motif ligand 8
IL: interleukin
IQR: interquartile range
KC-Like: keratinocyte chemoattractant-like
MCP-1: monocyte chemoattractant protein-1
NET: neutrophil extracellular trap
NSA: non-septic ascites
NT-proCNP: amino-terminal propeptide of C-type natriuretic peptide
PCT: procalcitonin
PRR: pattern recognition receptor
PT: prothrombin time
rcf: relative centrifugal force
ROC: receiver-operating characteristic
SIRS: systemic inflammatory response syndrome
SP: septic peritonitis.
