# Corrigendum: Biomarker Guided Diagnosis of Septic Peritonitis in Dogs

**DOI:** 10.3389/fvets.2020.00147

**Published:** 2020-03-19

**Authors:** Pia Martiny, Robert Goggs

**Affiliations:** College of Veterinary Medicine, Cornell University, Ithaca, NY, United States

**Keywords:** sepsis, biomarker, effusion, gradient, lactate

In the original article, there was a mistake in Tables 1–4 as published. The units for Procalcitonin (PCT) concentration were erroneously stated as ng/mL, but should be stated as pg/mL. The corrected Tables appear below.

**Table 1:** Comparisons of blood biomarker concentration with effusion biomarker concentrations in dogs with septic peritonitis (SP) and in dogs with non-septic peritonitis (NSA).

	**SP**				**NSA**			
	**Blood median IQR**	**Effusion median IQR**	***P*-value**	***P*_**corr**_**	**Blood median IQR**	**Effusion median IQR**	***P*-value**	***P*_**corr**_**
Glucose (mg/dL)	111	20	0.0011	**0.0121**	101	94	0.1648	1.8128
	81–124	20–108			79–118	78–99		
Glucose (mmol/L)	6.2	1.1	0.0011	**0.0121**	5.6	5.2	0.1648	1.8128
	4.5–6.9	1.1–6.0			4.4–6.6	4.4–5.5	0.1648	1.8128
Lactate (mmol/L)	2.7	7.9	0.0066	0.0726	2.0	1.8	0.6998	7.6978
	1.2–4.2	2.7–11.3			1.3–2.6	1.1–2.8		
cfDNA (ng/mL)	379	1085	0.0003	**0.0033**	410	470	0.7381	8.1191
	316–430	493–2723			316–629	269–1157		
Nucleosomes (AU)	0.35	13.1	0.0001	**0.0011**	0.6	2.5	0.0289	0.1848
	0.2–1.1	4.4–25.1			0.3–1.8	0.3–12.9		
NT–proCNP (pmol/L)	11.9	6.2	0.2460	2.910	3.9	2.3	0.1134	1.333
	5.6–42.1	1.9–25.7			2.3–12.0	1.6–10.3		
PCT (pg/mL)	99.6	38.4	0.0028	**0.0352**	93.5	98.9	0.6226	6.8486
	40.3–182.9	0.0–119.2			54.7–208.1	29.7–201.6		
CCL2 (pg/mL)	1274	16066	0.0001	**0.0011**	249	683	0.0001	**0.0011**
	282–2715	2508–96300			21–431	449–2225		
CXCL8 (pg/mL)	785.4	4325.0	0.0090	0.099	550.3	129.6	0.1901	2.0911
	495.6–1857.0	21.7–75000.0			51.8–1372.0	21.7–444.7		
IL-10 (pg/mL)	8.5	303.1	0.0012	**0.0132**	8.5	8.5	0.0312	0.3432
	8.5–106.4	147.6–1584.0			8.5–8.5	8.5–31.9		
IL-6 (pg/mL)	9.9	2775.0	0.0001	**0.0011**	3.7	37.9	0.0029	**0.0319**
	3.7–1573.0	363.5–11495.0			3.7–15.9	3.7–1918.0		
KC-Like (pg/mL)	164.1	1215.0	0.0011	**0.0121**	20.3	456.2	0.0001	**0.0011**
	8.0–1137.0	37.2–6973.0			5.3–71.5	62.1–757.0		

*Median and IQR concentrations of measured biomarkers in blood and effusion in patients with SP and NSA, and results of univariate comparisons of biomarker concentrations between fluid types with each group by Wilcoxon matched-pairs signed rank test. Biomarkers significantly different after correction for multiple comparisons (P <0.05) between blood and effusion within SP and NSA groups are indicated in bold typeface. AU, arbitrary unit; CCL2, C-C Motif Chemokine Ligand-2; cfDNA, cell-free DNA; CXCL8, chemokine C-X-C motif ligand 8; IL, interleukin; IQR, interquartile range; KC-Like, keratinocyte chemoattractant-like; NSA, non-septic ascites; NT-proCNP, amino-terminal propeptide of C-type natriuretic peptide; P_corr_, P-value corrected for multiple comparisons; PCT, procalcitonin; SP, septic peritonitis*.

**Table 2:** Comparisons of blood biomarker concentrations in dogs with septic peritonitis (SP) and non-septic peritonitis (NSA).

	**Blood**			
	**SP median IQR**	**NSA median IQR**	***P*-value**	***P*_**corr**_**
Glucose (mg/dL)	111	101	0.7927	8.7197
	81–124	79–118		
Glucose (mmol/L)	6.2	5.6	0.7927	8.7197
	4.5–6.9	4.4–6.6		
Lactate (mmol/L)	2.7	2.0	0.3698	4.0678
	1.2–4.2	1.3–2.6		
cfDNA (ng/mL)	379	410	0.3953	4.3483
	316–430	316–629		
Nucleosomes (AU)	0.35	0.60	0.2043	2.2473
	0.2–1.1	0.3–1.8		
NT-proCNP (pmol/L)	11.9	3.9	0.0399	0.4389
	5.6–42.1	2.3–12.0		
PCT (pg/mL)	99.6	93.5	0.7299	8.0289
	40.3–182.9	54.7–208.1		
CCL2 (pg/mL)	1274	249	0.0029	**0.0319**
	282–2715	21–431		
CXCL8 (pg/mL)	785.4	550.3	0.188	2.068
	495.6–1857.0	51.8–1372.0		
IL-10 (pg/mL)	8.5	8.5	0.008	0.088
	8.5–106.4	8.5–8.5		
IL-6 (pg/mL)	9.9	3.7	0.0680	0.748
	3.7–1573.0	3.7–15.9		
KC-Like (pg/mL)	164.1	20.3	0.0731	0.8041
	8.0–1137.0	5.3–71.5		

*Median and IQR concentrations of measured biomarkers in blood in patients with SP and NSA, and results of univariate comparisons of biomarker concentrations between groups by Mann-Whitney U-test. Biomarkers identified to be significantly different in blood compared between SP and NSA groups after correction for multiple comparisons (P <0.05) are indicated in bold typeface. AU, arbitrary unit; CCL2, C-C Motif Chemokine Ligand-2; cfDNA, cell-free DNA; CXCL8, chemokine C-X-C motif ligand 8; IL, interleukin; IQR, interquartile range; KC-Like, keratinocyte chemoattractant-like; NSA, non-septic ascites; NT-proCNP, amino-terminal propeptide of C-type natriuretic peptide; P_corr_, P value corrected for multiple comparisons; PCT, procalcitonin; SP, septic peritonitis*.

**Table 3:** Comparisons of effusion biomarker concentrations in dogs with septic peritonitis (SP) and non-septic peritonitis (NSA).

	**Effusion**			
	**SP median IQR**	**NSA median IQR**	***P*-value**	***P*_**corr**_**
Glucose (mg/dL)	20	94	0.011	0.121
	20–108	78–99		
Glucose (mmol/L)	1.1	5.2	0.011	0.121
	1.1–6.0	4.3–5.5		
Lactate (mmol/L)	7.9	1.8	0.0001	**0.0011**
	2.7–11.3	1.1–2.8		
cfDNA (ng/mL)	1085	470	0.0205	0.2255
	493–2723	269–1157		
Nucleosomes (AU)	13.1	2.5	0.0056	0.0616
	4.4–25.1	0.3–12.9		
NT-proCNP (pmol/L)	6.2	2.3	0.2806	3.0866
	1.9–25.7	1.6–10.3		
PCT (pg/mL)	38.4	98.9	0.0489	0.5379
	0–119.2	29.7–201.6		
CCL2 (pg/mL)	16066	683	0.0011	**0.0121**
	2508–96300	449–2225		
CXCL8 (pg/mL)	4325.0	129.6	0.0113	0.1243
	21.7–75000.0	21.7–444.7		
IL-10 (pg/mL)	303.1	8.5	0.0001	**0.0011**
	147.6–1584.0	8.5–31.9		
IL-6 (pg/mL)	2775.0	37.9	0.0011	**0.0121**
	363.5–11495.0	3.7–1918.0		
KC-Like (pg/mL)	1215.0	456.2	0.312	3.432
	37.2–6973.0	62.1–757.0		

*Median and IQR concentrations of measured biomarkers in effusion in patients with SP and NSA, and results of univariate comparisons of biomarker concentrations between groups by Mann-Whitney U-test. Biomarkers identified to be significantly different in effusion compared between SP and NSA groups after correction for multiple comparisons (P <0.05) are indicated in bold typeface. AU, arbitrary unit; CCL2, C-C Motif Chemokine Ligand-2; cfDNA, cell-free DNA; CXCL8, chemokine C-X-C motif ligand 8; IL, interleukin; IQR, interquartile range; KC-Like, keratinocyte chemoattractant-like; NSA, non-septic ascites; NT-proCNP, amino-terminal propeptide of C-type natriuretic peptide; P_corr_, P value corrected for multiple comparisons; PCT, procalcitonin; SP, septic peritonitis*.

**Table 4:** Comparisons of blood-effusion biomarker concentration gradients in dogs with septic peritonitis (SP) and non-septic peritonitis (NSA).

	**Blood-Effusion Gradient**			
	**SP median IQR**	**NSA median IQR**	***P*-value**	***P*_**corr**_**
Glucose (mg/dL)	59	6	0.0009	**0.0099**
	16–91	−6–27		
Glucose (mmol/L)	3.3	0.3	0.0009	**0.0099**
	0.9–5.1	−0.3–1.5		
Lactate (mmol/L)	−4.9	0.2	0.0015	**0.0165**
	−8.5 to −0.5	−0.9–0.6		
cfDNA (ng/mL)	−757	−31	0.005	0.055
	−2331 to −179	−527–165		
Nucleosomes (AU)	−13.0	−0.7	0.0066	0.0726
	−22.5 to −3.4	−12.8–0.3		
NT-proCNP (pmol/L)	2.4	0.8	0.3744	4.1624
	−1.6–7.4	−0.5–2.6		
PCT (pg/mL)	27.9	22.4	0.0861	0.9471
	8.3–98.7	−32.7–33.7		
CCL2 (pg/mL)	−14151	−561	0.0006	**0.0066**
	−91817 to −2243	−1827 to −110		
CXCL8 (pg/mL)	−3657.0	134.1	0.0129	0.1419
	−68631.0–623.7	−51.1–1316.0		
IL-10 (pg/mL)	−294.6	0.0	0.0004	**0.0044**
	−1430.0 to −63.8	−23.4 to 0.0		
IL-6 (pg/mL)	−2772.0	−0.5	0.0009	**0.0099**
	−10272.0 to −359.8	−1914.0 to 0.0		
KC-Like (pg/mL)	−937.3	−425.5	0.3786	4.1646
	−4615.0 to −31.9	−716.7 to −41.3		

*Median and IQR blood-effusion concentration gradients of measured biomarkers in patients with SP and NSA, and results of univariate comparisons of biomarker concentration gradients between groups by Mann-Whitney U-test. Biomarker bloodeffusion gradients identified to be significantly different compared between SP and NSA groups after correction for multiple comparisons (P <0.05) are indicated in bold typeface. AU, arbitrary unit; CCL2, C-C Motif Chemokine Ligand-2; cfDNA, cell-free DNA; CXCL8, chemokine C-X-C motif ligand 8; IL, interleukin; IQR, interquartile range; KC-Like, keratinocyte chemoattractant-like; NSA, non-septic ascites; NT-proCNP, amino-terminal propeptide of C-type natriuretic peptide; P_corr_, P value corrected formultiple comparisons; PCT, procalcitonin; SP, septic peritonitis*.

The authors apologize for this error and state that this does not change the scientific conclusions of the article in any way. The original article has been updated.

